# HTR1D functions as a key target of HOXA10-AS/miR-340-3p axis to promote the malignant outcome of pancreatic cancer via PI3K-AKT signaling pathway

**DOI:** 10.7150/ijbs.70546

**Published:** 2022-05-27

**Authors:** Wu Wu, Qujin Li, Zhu Zhu, Chunming Li, Peilin Lu, Xi Zhou, Yujing Huang, Yan Liu, Menghao Wang, Jianping Gong

**Affiliations:** 1 Department of Hepatobiliary Surgery, The Second Affiliated Hospital of Chongqing Medical University, Chongqing, China; 2 Department of Dermatology, The First Affiliated Hospital of Chongqing Medical University, Chongqing, China; 3 Department of Gastroenterology, Affiliated Hospital of Southwest Medical University, Luzhou, Sichuan, China; 4 Department of Gastroenterology, The Fifth People's Hospital of Chengdu, Chengdu, Sichuan, China

**Keywords:** pancreatic cancer, ceRNA, HTR1D, HOXA10-AS, miR-340-3p

## Abstract

Competing endogenous RNAs (ceRNAs) are a newly discovered class of molecular regulators involved in many diseases, especially tumors. Therefore, exploration of the potential ceRNA regulatory network regarding the occurrence and development of pancreatic cancer will provide a new theoretical basis for its diagnosis and treatment. Based on the above background, we applied a bioinformatics approach to mine the public database The Cancer Genome Atlas (TCGA) and performed a series of subsequent molecular biology assays to confirm the hypothesis that HOXA10-AS/ miR-340-3p/HTR1D axis could modulate the malignant progression of pancreatic cancer. Here, our present study demonstrated that the expression level of HTR1D, positively correlated with the level of lncRNA HOXA10-AS and negatively associated with the level of miR-340-3p, was significantly increased in pancreatic cancer cell lines (PCs) compared with that in normal HPDE6-C7 cells. Knocking down HTR1D obviously inhibited the proliferation and migration of PCs and promoted apoptosis by upregulating p-AKT. Elevated miR-340-3p blocked the progression of pancreatic cancer by downregulating HTR1D. Lessened level of lncRNA HOXA10-AS reduced the sponging of miR-340-3p, resulting in an increase of miR-340-3p and a subsequent decrease of HTR1D to ultimately suppress the malignant biological behaviors of cancer. These data illustrated that the HOXA10-AS/miR-340-3p/HTR1D ceRNA axis acted a crucial part in the malignant biological behavior of pancreatic cancer in an AKT-dependent manner.

## Introduction

Pancreatic cancer is a lethal disease worldwide which has a KRAS mutation rate as high as 90% 1. Pancreatic cancer is characterized by early metastasis during the disease progression period and a lack of effective therapeutic methods, which leads to an extremely poor prognosis and a low survival rate 2-5. Resistance to chemotherapy plays an imperative role in its lethality, and surgery is known as the only existing curative treatment for pancreatic cancer 6, 7. Therefore, it is essential to investigate accurate and predictable biomarkers to diagnose pancreatic cancer at an earlier stage.

5-Hydroxytryptamine (5-HT or serotonin) is a neurotransmitter involved in numerous neurological processes of cellular signals transduction and cell growth maintenance 8-10. In addition, 5-HT receptors (5-HTRs) serve vital roles in these processes by binding to the surface of cell membrane 11. According to recent studies, 5-HTRs were linked to malignant tumors, such as melanoma 12, breast cancer 13, 14, lung cancer 15 and colon cancer 16. For example, 5-HTR1B, 5-HTR3 and 5-HTR4 were proven to promote the proliferation of colorectal cancer cells (CRCs) 17. Therefore, 5-HTRs could be potential clinical targets of cancer therapies. HTR1D is one of 14 types of HTRs, and only a few studies investigated its roles in tumors. The extracts of the traditional Chinese medicine Zuojin Wancan were reported to influence the biological behavior of CRCs by regulating HTR1D 8. At present, there are no research reports on HTR1D in the context of pancreatic cancer. Therefore, exploring the role of HTR1D in pancreatic cancer may provide a novel molecular therapeutic target.

Noncoding RNA (ncRNA) refers to a type of RNA that does not encode protein, including rRNA, long noncoding RNA (lncRNA), microRNA (miRNA) and so on. Even if ncRNAs can't encode proteins, the regulatory network regarding them contains many molecular targets, so ncRNAs were considered as key regulators in many physiological processes 18. Among them, lncRNAs and miRNAs are the most extensively studied ncRNAs that have been confirmed to be associated with various biological processes, such as transcription regulation, cell proliferation, chromatin remodeling, and even tumorigenesis 19-21. In pancreatic cancer, ncRNAs also have been identified as important tumor drivers or suppressors 22. The ceRNAs have recently attracted much attention from the academia, because they represent a novel mode of gene expression regulation 23. Mechanistically, ceRNAs (miRNA, lncRNA, pseudogene, etc.) can compete with the same miRNA via miRNA response elements (MREs) to alter the expression level of targets 24, 25. For instance, a recent study showed that lncRNA PVT1, served as a ceRNA, could promote gemcitabine resistance in pancreatic cancer by activating the Wnt/β-catenin and autophagy pathways through modulating the miR-619-5p/Pygo2 and miR-619-5p/ATG14 axes 26. Another work found that lncRNA GSTM3TV2 could function as a ceRNA to upregulate LAT2 and OLR1 through competitively sponging let-7, which promoted gemcitabine resistance in pancreatic cancer 27. Moreover, Yushui Ma et al. claimed that the lncRNA NORAD, a novel ceRNA, could promote the proliferation and self-renewal of pancreatic cancer stem cells 28. Nevertheless, only a small number of ncRNAs have been revealed to be relevant to pancreatic cancer, and more additional ncRNAs, which can precisely predict the occurrence and progression of pancreatic cancer, should be discovered.

In this work, we identified three new molecular targets, namely HTR1D, HOXA10-AS and miR-340-3p. Fortunately, none of these molecules have been previously reported in pancreatic cancer. Therefore, an in-depth understanding of their roles in the tumorigenesis of pancreatic cancer will provide new strategies for clinical diagnosis and treatment.

## Materials and methods

### Cell culture

Six human PCs (BxPC-3, SW1990, PANC-1, CFPAC-1, AsPC-1, and T3M4) and the nonmalignant pancreatic ductal epithelial cell line HPDE6-C7 were obtained from Peking University School of Medicine. The cells were cultured in high-glucose compete DMEM supplemented with 10% FBS (Gibco, USA) at 37°C in an atmosphere of 5% CO_2_ in a constant humidity incubator.

### RNA extraction and quantitative real time-PCR assay

Total RNA was extracted from the tumor cells and tissues using TRIzol (Sigma) reagent according to a previously described protocol 29. A NanoDrop 2000 (Thermo, USA) was used to quantify the concentrations of the RNA. Total RNA was then reverse transcribed to complementary DNA (cDNA) using a miScript II RT kit (TaKaRa Biotechnology Co., Ltd., Dalian, China), and qPCR was performed using TB Green® Premix Ex Taq™ (RR820A, Takara, Japan) and a Bio-Rad real-time PCR instrument.

The primer sequences are listed in **Supplementary Table 1**.

### Western blotting (WB)

Protein samples were prepared by lysis of the cells and tissues in RIPA buffer and subsequent centrifugation. A bicinchoninic acid (BCA) protein assay kit (Thermo Fisher) was used to quantify the protein concentrations according to the manufacturer's protocol. SDS-PAGE (10% or 12.5%) was used to separate 20 µg of protein per sample, and the proteins were transferred onto the nitrocellulose (NC) membranes at a constant voltage of 80 V for 2 h. Then, 5% skim milk was used to block the NC membranes for 1 h, and the membranes were incubated with primary antibodies at 4°C overnight. The NC membranes were washed three times with Tris-buffered saline containing 0.1% Tween 20 (TBST), and incubated with horseradish peroxidase-conjugated secondary antibodies at room temperature for 1 h. Specific bands were detected using a Bio-Rad chemiluminescence imaging system. The following antibodies were used: anti-HTR1D (ABP57360, Abbkine), anti-Bax (ab32503, Abcam), anti-Bcl-2 (ab32124, Abcam), anti-p-AKT (4060S, Cell Signaling), and anti-AKT (4685S, Cell Signaling).

### Transwell assay

The migration ability of PANC-1 and CFPAC-1 cells was assessed by the Transwell assays. Serum-free DMEM (300 μl) was added to each Transwell chamber, and 1×10^4^ PANC-1 and CFPAC-1 cells in the logarithmic growth phase were inoculated in every chamber. Next, 500 μl of 10% complete DMEM was added to the wells of a 24-well plate, and the samples were incubated in a constant temperature incubator at 37°C and 5% CO_2_ for 12 h. Then, 500 μl of paraformaldehyde was added for 30 min to fix the cells. Afterward, 500 μl of crystal violet dye (Beyotime, China) was added for 15 min to stain the cells. Finally, the images were acquired after complete drying of the Transwell chambers.

### Wound healing assay

Initially, a marker pen was used to draw a series of lines at the intervals of 1 cm on the exterior bottom surface of a 6-well plate across all wells to ensure that the area of each well contained at least 5 lines. Then, 5×10^5^ cells were added to each well and incubated in a constant temperature incubator at 37°C until the cells were confluent. The next day, two parallel lines were scratched using a 10 µl pipette tip positioned at a 90º angle to the markings. Then, the cells were washed 3 times with PBS to remove detached cells, and the cells were incubated in serum-free medium in an incubator at 37°C and 5% CO_2_. The wells were monitored, and the images were acquired using an inverted microscope after 0, 24, and 48 h.

### Flow cytometry

Apoptosis was detected using a kit (C1062L, Beyotime). PANC-1 and CFPAC-1 cells were seeded and cultured in 6-well plates, and the cells at a density of 80% were digested by trypsin to generate a single cell suspension (1×10^6^ cells/ml). After cell suspension was centrifuged to remove the supernatant (1,000 g, 5 min), 195 μl of Annexin V-fluorescein isothiocyanate (FITC) binding solution was used to resuspend the cells. Then, 5 µl of Annexin V-FITC and 10 µl of propidium iodide (PI) staining solutions were added to this cell suspension, and the samples were incubated at room temperature in the dark for 20 min. The results were obtained by flow cytometry (FITC signal corresponded to green fluorescence, and the PI signal was registered as red fluorescence).

### Cell Counting Kit-8 (CCK-8), EdU, and colony formation assays

A total of 5×10^3^ cells/well in the logarithmic growth phase were seeded into a 96-well plate approximately 24 h prior to the transfections or treatments. Then, 10 μl of CCK-8 reagent (Bimake, USA) was added to each well for 1-4 h (37°C and 5% CO_2_). The absorbance was detected at 450 nm using a microplate autoreader to evaluate relative cell viability. Briefly, all EdU staining procedures were performed according to the manufacturer's instructions as described previously 29. For the colony formation assay, 1×10^3^ cells/well in the logarithmic growth phase were seeded into a 6-well plate approximately 24 h prior to the transfections or treatments and cultured in complete DMEM containing 10% FBS in a stable environment (37°C and 5% CO_2_) for approximately 2 weeks. Finally, the cells were removed from the incubator, fixed, stained, and imaged.

### Dual-luciferase reporter assay

The potential binding targets of miR-340-3p (HTR1D and HOXA10-AS containing miR-340-3p binding sites and their corresponding mutants without miR-340-3p binding sites) were subcloned into the 3' untranslated region (UTR) of R-luciferase (Renilla) in the psiCHECK2 luciferase reporter vector. Then, all the constructed plasmids above were cotransfected into cells with miR-340-3p. All fluorescent activity detection procedures were performed according to the manufacturer's instructions of the Dual Luciferase Assay Kit (RG027, Beyotime, China).

### Immunohistochemical staining

A human pancreatic cancer tissue microarray used in the present study was purchased from Shanghai Xinchao Biotechnology Co., Ltd. (China), and the fragments of mouse subcutaneous tumor tissue were embedded and sectioned by the laboratory technician Hongqiang Yu. The paraffin-embedded sections were placed in an oven at 65°C for 30 min; then, the sections were immersed in antigen retrieval solution, dewaxed, and transferred into a microwave oven set on high power for two rounds of 8-min incubation. Next, the sections were treated with 3% hydrogen peroxide and methanol for 30 min at room temperature. The samples were blocked with goat serum and incubated with a primary antibody at 4°C overnight. The samples were treated with a secondary antibody, and 3,3'-diaminobenzidine (DAB) was used as a chromogenic agent to evaluate the samples using a microscope. Finally, hematoxylin reagent was added to stain the nuclei, and the samples were sealed using neutral gum. The following antibodies were used: anti-HTR1D (ABP57360, Abbkine, 1:200) and anti-KI67 (12202S, Cell Signaling, 1:500).

### Subcutaneous tumor xenograft model in nude mice

The proliferation ability of tumor cells was assessed in vivo in a subcutaneous tumor xenograft model in nude mice. All animal experiments were approved by the Animal Ethics Committee of Chongqing Medical University. Male nude mice aged 4-6 weeks were purchased from Jicui Yaokang Biotechnology Co., Ltd. (Jiangshu, China). A total of 3×10^6^ PANC-1 cells and 5×10^6^ CFPAC-1 cells with stable expression of the corresponding plasmids were suspended in 100 μl of phosphate-buffered saline (PBS) and injected subcutaneously into the right armpit of nude mice. Then, the mice were fed and housed under normal conditions. After approximately 2-3 weeks, the tumor masses were removed, weighed, imaged, and prepared for subsequent experiments according to the growth status of the tumors.

### Statistical analysis

All the data were presented as the mean±standard deviation, and the experiments were repeated at least three times independently. The differences were analyzed using 2-tailed t tests. Multiple-group comparisons were performed by two-way analysis of variance (ANOVA). All analyses were performed using SPSS 26 for Windows (SPSS, Inc., Chicago, IL, USA) and GraphPad Prism (Version 6.0) for Windows (GraphPad Software, Inc., San Diego, CA, USA). P≤0.05 was considered to indicate statistical significance (^*^p<0.05; ^**^p<0.01; ^***^p<0.001; n.s., not significant).

## Results

### HTR1D was identified by TCGA database analysis

**Fig 1:**
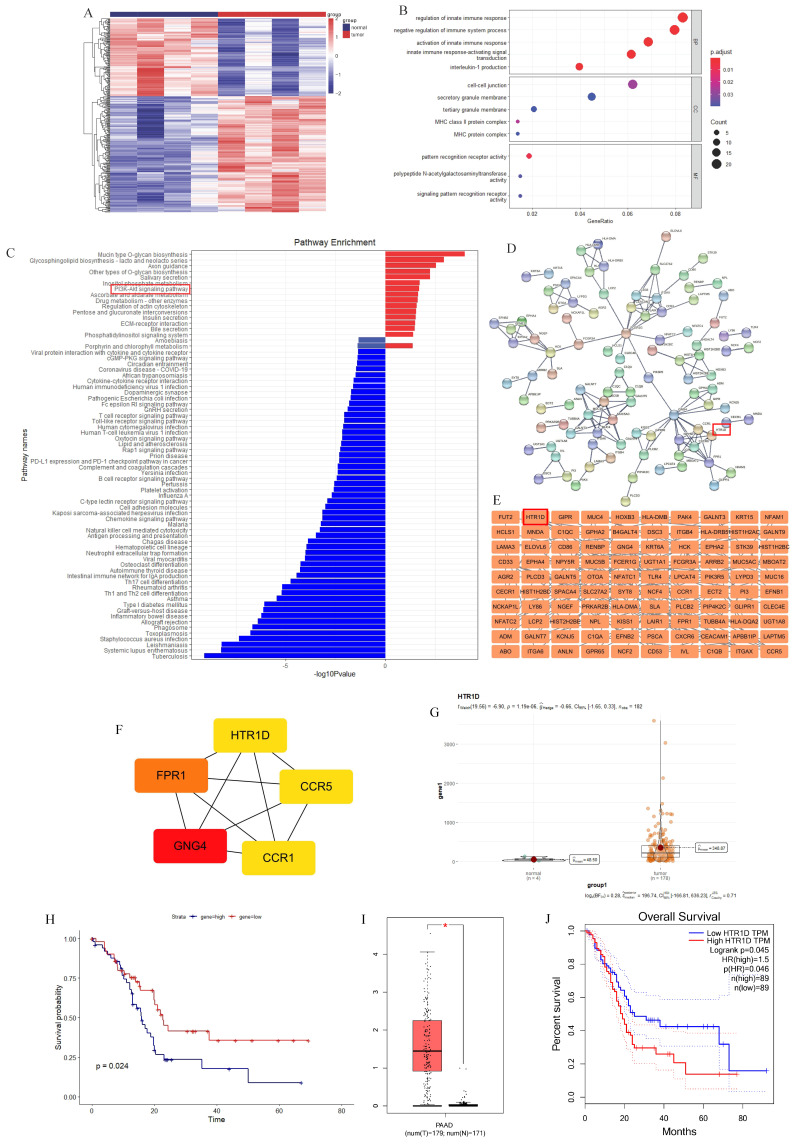
Screening for the target gene (HTR1D). **a** The heatmap for differentially expressed mRNAs of paired pancreatic cancer in TCGA database. **b-c** GO (b) and KEGG (c) analysis for differentially expressed mRNAs. **d-e** PPI network for differentially expressed mRNAs. **f** 5 hub genes of differentially expressed mRNAs. **g-h** The expression difference and survival analysis curve of HTR1D using RStudio based on the bioinformatics analysis of TCGA data. **i-j** The expression difference and survival analysis curve of HTR1D based on GEPIA online website.

To discover more additional biomarkers for pancreatic cancer with diagnostic and therapeutic significance, we downloaded pancreatic cancer data from the official TCGA website and performed relevant bioinformatics analysis. We performed a two-way hierarchical clustering analysis of mRNA data, and the results showed that there were significant differences between tumor samples and their paired normal samples (Fig. 1a). We obtained 191 upregulated genes and 128 downregulated genes (data not shown). Then, we performed Gene Ontology (GO) and Kyoto Encyclopedia of Genes and Genomes (KEGG) enrichment analysis on these differentially expressed genes (DEGs), and the results revealed that the PI3K-AKT signaling pathway was significantly enriched in the upregulated genes (Fig. 1c). To screen out target genes, we used the Search Tool for the Retrieval of Interacting Genes/Proteins (STRING) online website and Cytoscape software to construct a protein interaction network of DEGs and then utilized the cytoHubba plug-in to calculate 5 hub genes (CCR1, CCR5, GNG4, FPR1, and HTR1D), as shown in Fig. 1d-f. Then, we analyzed the prognostic value of these 5 hub genes separately, and found that only HTR1D was a statistically significant prognostic indicator (Fig. 1g-h, Fig. S1a-d), so we focused our studies on HTR1D. Finally, we applied the Gene Expression Profiling Interactive Analysis (GEPIA) website to verify the prediction results. The results showed that HTR1D, highly expressed in pancreatic cancer, was a significant indicator of patient survival (Fig. 1i-j).

### HTR1D promoted the proliferation and migration of PCs in vitro

**Fig 2:**
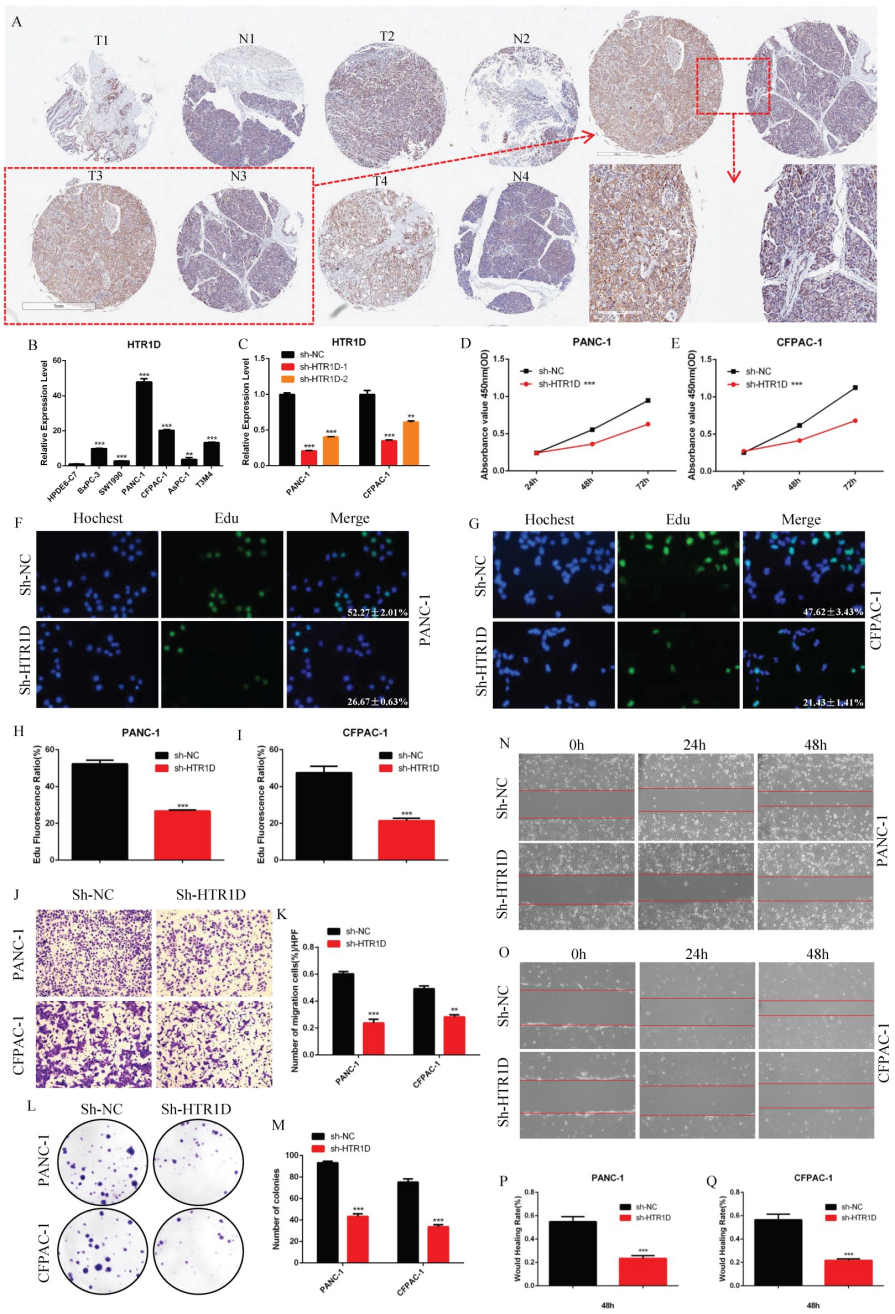
HTR1D promotes proliferation and migration of PCs in vitro. **a** The expressed level of HTR1D in 4 random pairs among 90 pairs of pancreatic cancer and paracancerous tissue microarrays. **b** The mRNA Expression level of HTR1D in different PCs. **c** The efficiency of two knockdown sequences was tested by qPCR in PANC-1 and CFPAC-1. **d-e** The absorbance at 450nm was measured to evaluate the effect on cell viability by CCK8. **f-g** EdU incorporation assay was performed to detect the proliferation of PANC-1 (f) and CFPAC-1 (g). **h-i** Quantification of the data shown in f and g. **j-k** Representative images of the migration ability of PANC-1 and CFPAC-1 by Transwell assay. **l-m** Representative images of colony formation assay for PANC-1 and CFPAC-1. **n-o** Representative images of wound healing assay for PANC-1 (n) and CFPAC-1 (o). **p-q** Quantification of the data shown in n and o. Each experiment was performed three times independently and results are presented as mean ± s.d. Student's t-test was used to analyze the data. (^*^p < 0.05; ^**^p < 0.01; ^***^p < 0.001)

To deeply understand the function of HTR1D in pancreatic cancer in detail, we also performed a series of in vitro and in vivo experiments. We purchased a human pancreatic cancer tissue chip from Shanghai Xinchao Company and performed an immunohistochemical experiment. The results illustrated that HTR1D was expressed at a high level in pancreatic cancer tissues compared to that in the normal pancreatic tissues (Fig. 2a). For the sake of further exploring the specific underlying mechanism of HTR1D in pancreatic cancer, we used qPCR to detect the mRNA expression level of HTR1D in different PCs. The results showed that HTR1D was elevated in PCs, and that the HTR1D acquired the highest mRNA expression level in PANC-1 and CFPAC-1 cells among all PCs, so we selected PANC-1 and CFPAC-1 for subsequent experimental verification (Fig. 2b). Then, we constructed two HTR1D knockdown sequences (sh-HTR1D-1 and sh-HTR1D-2). The subsequent experiments showed that both sequences exhibited obvious effects, but the knockdown efficiency of sh-HTR1D-1 was better (Fig. 2c). In addition, we performed several other functional assays to verify the effect of HTR1D on PCs in vitro. The CCK-8 assay results demonstrated that lessened HTR1D had a lower OD value at 450nm compaired to that in the control group (Fig. 2d-e). EdU results showed that knocking down HTR1D could significantly reduce the EdU fluorescence ratio (Fig. 2f-i). The results of Transwell and colony formation experiments descriped that decreased HTR1D significantly reduced the migration rate and colonies of PCs (Fig. 2j-m). Furthermore, the results of the wound healing assay showed that the wound healing degree of the sh-HTR1D group was significantly decreased at the corresponding time points (Fig. 2n-q).

### HTR1D inhibited the apoptosis of PCs in vitro and promoted the proliferation in vivo

**Fig 3:**
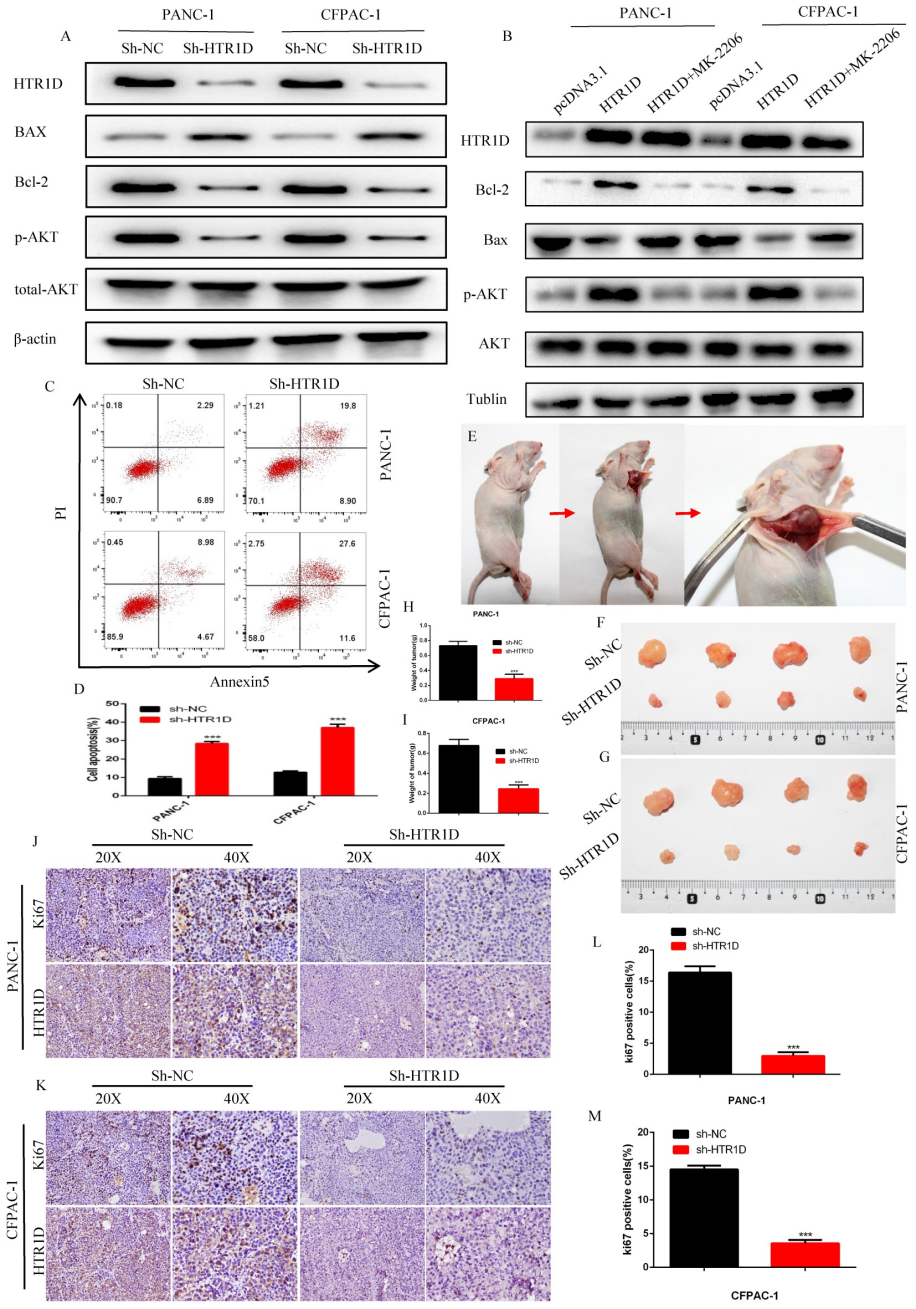
HTR1D inhibits apoptosis in vitro and promotes proliferation in vivo. **a-b** Western blot was performed to detect apoptosis-related proteins and phosphorylated AKT with or without MK-2206 (AKT inhibitor) in each cell line transfected with designated plasmids. **c-d** Flow cytometry analysis was applied to test cell apoptosis for PANC-1 and CFPAC-1. **e** Schematic diagram of subcutaneous tumor formation. **f-g** Tumors derived from each cell line were displayed. **h-i** Quantification of tumor weight from f and g. **j-k** Representative images of immumohistochemical staining of tumors from f and g. **l-m** Quantification of the data shown in j and k. Each experiment was performed three times independently and results are presented as mean ± s.d. Student's t-test was used to analyze the data. (^*^p < 0.05; ^**^p < 0.01; ^***^p < 0.001)

To further clarify the function of HTR1D, we next used WB and flow cytometry to detect apoptosis in PCs. After knocking down HTR1D, the proapoptotic gene Bax was upregulated, while the apoptosis-suppressing gene Bcl-2 was downregulated (Fig. 3a). Meanwhile, we also found that the phosphorylation level of AKT, a key gene in the PI3K-AKT signaling pathway, was significantly reduced. Interestingly, we reversed the effects of HTR1D on pancreatic cancer using MK-2206, indicating that the effect of HTR1D on pancreatic cancer, at least in part, was mediated by the PI3K-AKT signaling pathway (Fig. 3b). The results of flow cytometry experiments showed that knocking down HTR1D caused a significant increase in the proportion of apoptotic cells in PCs (Fig. 3c-d). We also generated a mouse subcutaneous tumor model to observe the effect of HTR1D on the proliferation of PCs in vivo (Fig. 3e). We found that both tumor size and tumor weight were significantly decreased second to the reduced HTR1D (Fig. 3f-i). What's more, we detected the Ki67 proliferation index of tumor samples in immunohistochemical experiments, and the results suggested that Ki67 was significantly reduced with the decreased HTR1D (Fig. 3j-m). In summary, HTR1D could affect the proliferation, migration and apoptosis of pancreatic cancer partly via the PI3K-AKT signaling pathway.

### Hsa-miR-340-3p, which was negatively correlated with HTR1D in PCs, could directly targeted HTR1D and inhibited the expansion of PCs in vitro

**Fig 4:**
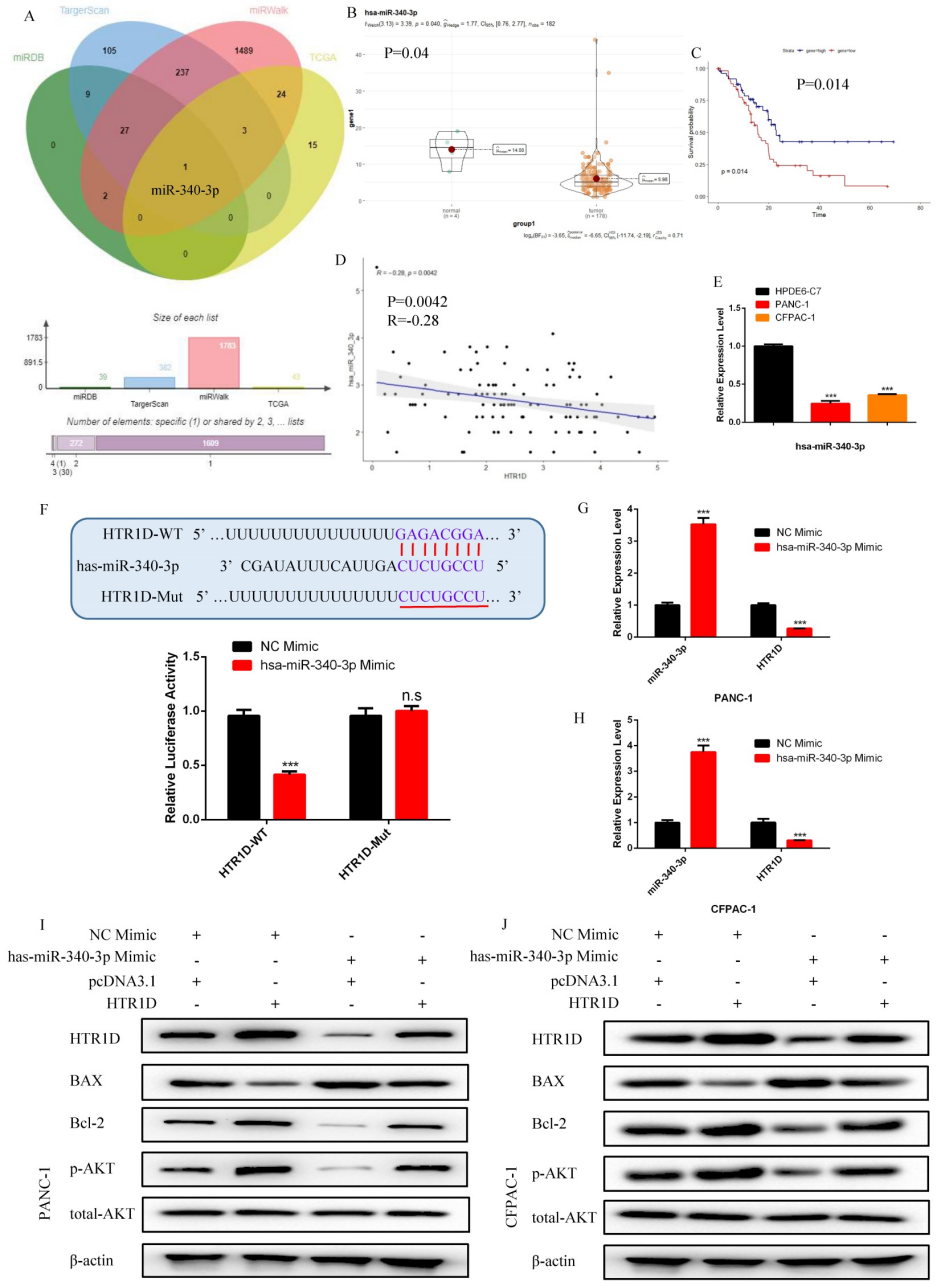
hsa-miR-340-3p is negatively correlated with the expression of HTR1D in pancreatic cancer, and promotes the apoptosis of PCs in vitro. **a** The venn diagram for the intersection of the upstream regulatory miRNAs of HTR1D predicted by different databases and the differentially expressed miRNAs of liver cancer samples in TCGA database. **b-c** The expression difference and survival analysis curve of hsa-miR-340-3p using RStudio based on the bioinformatics analysis of TCGA data. **d** Correlation analysis of the expression of HTR1D and hsa-miR-340-3p in TCGA pancreatic cancer samples. **e** The mRNA expression level of hsa-miR-340-3p was detected by qPCR in PANC-1 and CFPAC-1. **f** The dual luciferase reporter gene assay was performed to verify the interaction between HTR1D and hsa-miR-340-3p. **g-h** The effect of overexpressing hsa-miR-340-3p on the expression level of HTR1D in each cell line by qPCR. **i-j** Western blot was performed to detect apoptosis-related proteins and phosphorylated AKT in each cell line transfected with designated plasmids. Each experiment was performed three times independently and results are presented as mean ± s.d. Student's t-test was used to analyze the data. (^*^p < 0.05; ^**^p < 0.01; ^***^p < 0.001)

In order to ulteriorly excavate the upstream regulatory ceRNA network of HTR1D, we utilized relevant databases to screen candidate targets and finally identify miR-340-3p as the only target (Fig. 4a). Based on the analysis of differential expression, survival and correlations, the expression level of miR-340-3p was decreased in PCs (Fig. 4b) and was negatively correlated with HTR1D (Fig. 4d). We also found that miR-340-3p had a significant impact on the survival of patients (Fig. 4c). To dig deeper into the role of miR-340-3p in pancreatic cancer, we detected its mRNA expression level in PANC-1 and CFPAC-1 cells. As shown in Fig.4e, miR-340-3p was downregulated in PCs, which was consistent with our previous assumptions. To determine the interaction between miR-340-3p and HTR1D, we performed a dual luciferase reporter gene experiment, and the results showed that miR-340-3p could indeed act on HTR1D followed by causing a remarkable decrease of fluorescence activity (Fig. 4f). Moreover, we also transfected miR-340-3p mimics into PCs and detected the expression level of HTR1D by qPCR. The results showed that miR-340-3p could significantly inhibit the expression of HTR1D in mRNA level (Fig. 4g-h), indicating that miR-340-3p regulates HTR1D by promoting mRNA degradation rather than inhibiting protein translation.

**Fig 5:**
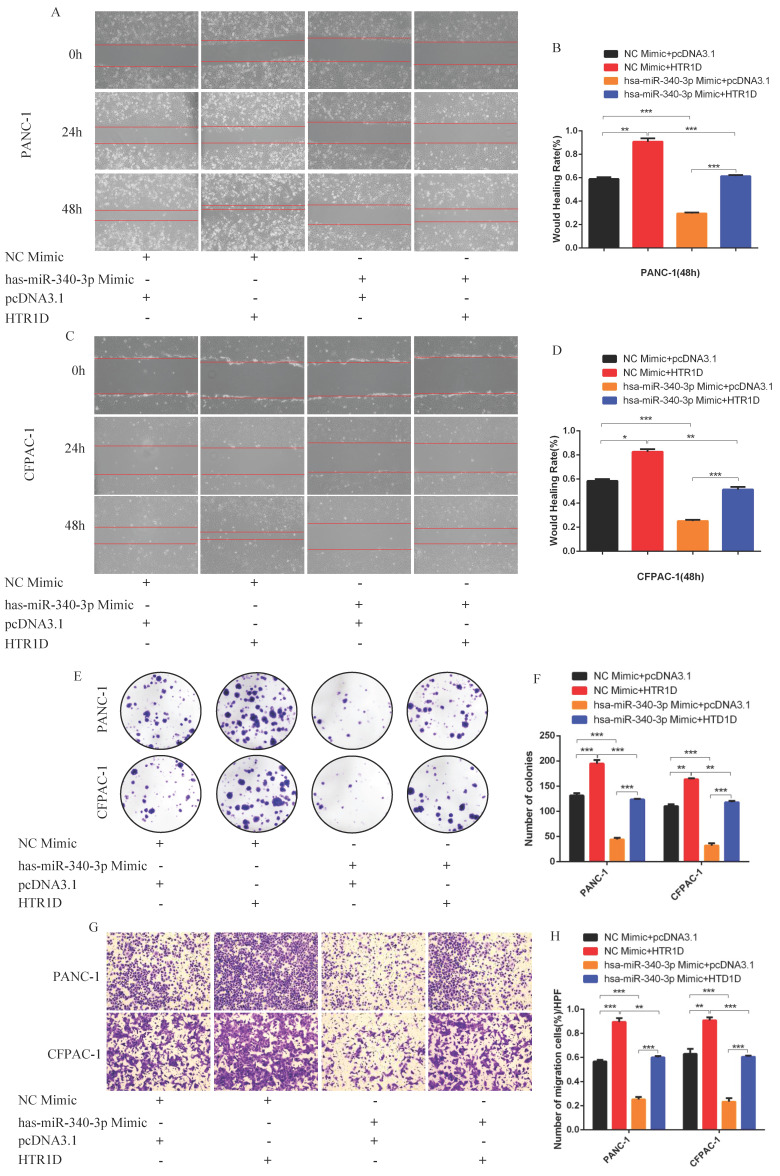
hsa-miR-340-3p inhibits the migration of pancreatic cancer cells in vitro. **a-d** Representative images of wound healing assay for two cell lines transfected with corelated plasmids. b and d revealed quantification of the data shown in a and c. **e-f** Representative images of colony formation assay for PANC-1 and CFPAC-1. **g-h** Representative images of the migration ability of PANC-1 and CFPAC-1 by Transwell assay. Each experiment was performed three times independently and results are presented as mean ± s.d. Student's t-test was used to analyze the data. (^*^p < 0.05; ^**^p < 0.01; ^***^p < 0.001)

**Fig 6:**
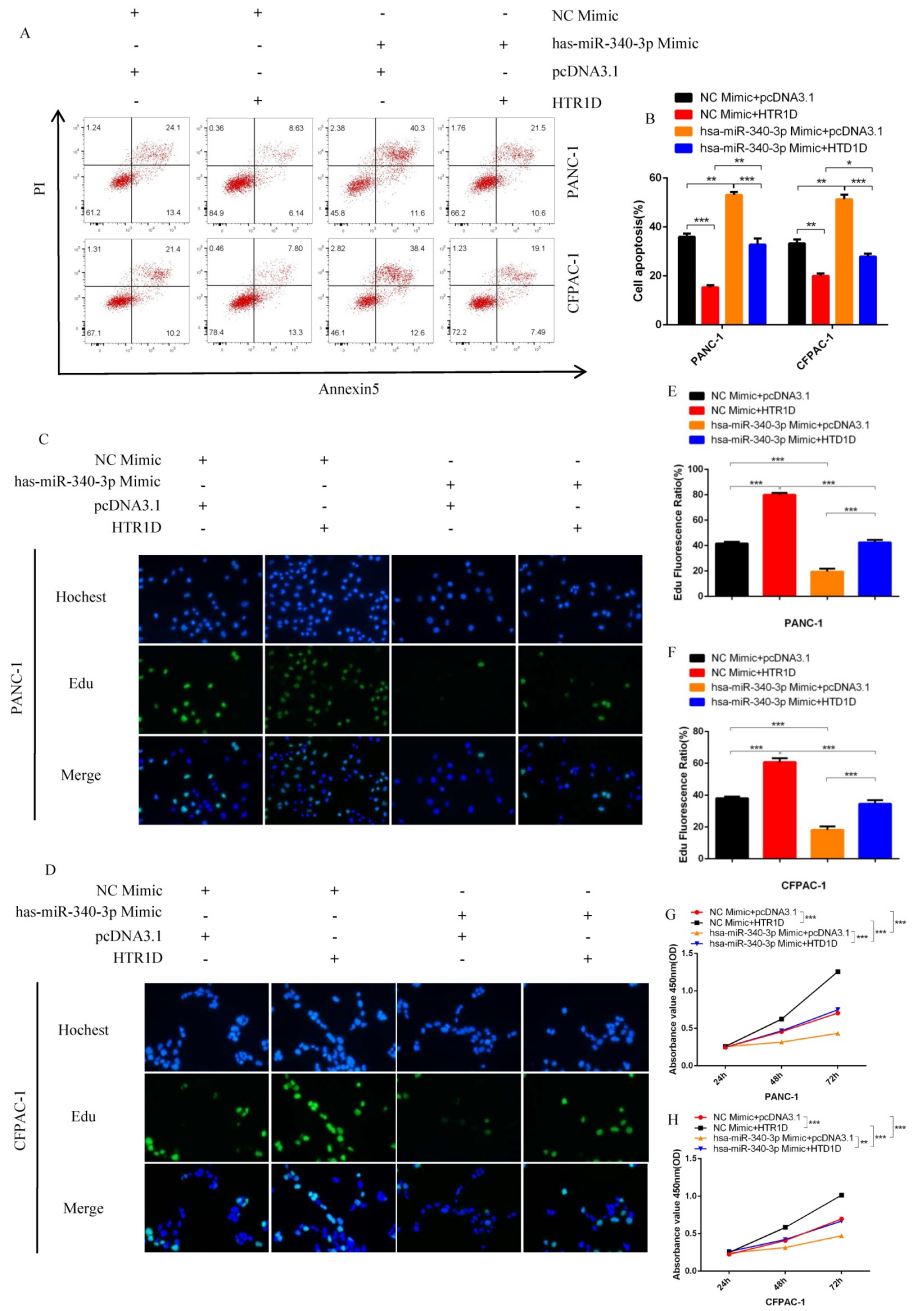
hsa-miR-340-3p inhibits the proliferation of PCs in vitro. **a-b** Flow cytometry analysis was applied to detect cell apoptosis for two cell lines transfected with corelated plasmids. b revealed quantification of the data shown in a. **c-d** EdU incorporation assay was performed to detect the proliferation of PANC-1 (c) and CFPAC-1 (d). **e-f** Quantification of the data shown in c and d. **g-h** CCK8 was performed to evaluate the cell viability of PANC-1 (g) and CFPAC-1 (h). Each experiment was performed three times independently and results are presented as mean ± s.d. Student's t-test was used to analyze the data. (^*^p < 0.05; ^**^p < 0.01; ^***^p < 0.001)

For purpose of proving that miR-340-3p can affect the progression of pancreatic cancer through modulating HTR1D, we cotransfected cells with related plasmids and performed a series of functional phenotyping assays. The WB results showed that the overexpression of miR-340-3p could promote apoptosis, but increased HTR1D could antagonize the effect of elevated miR-340-3p on PCs (Fig. 4i-j). The results of wound healing assays illustrated that the upregulated miR-340-3p could significantly inhibit the healing rate of PCs and that elevated HTR1D could reverse the above effect (Fig. 5a-d). The results of colony formation assays showed that enhancive miR-340-3p could significantly reduce the number of colonies, while raised HTR1D could attenuation the above phenomenon (Fig. 5e-f). The results of Transwell experiments demonstrated that upregulating the expression of miR-340-3p could obviously decrease the migration rate of PCs, while increased HTR1D could neutralize the above inhibitory effect of miR-340-3p on cellular migration (Fig. 5g-h). Moreover, the flow cytometry, EdU and CCK-8 results were all consistent with the above experimental results (Fig. 6). In short, here we found that miR-340-3p inhibited the progression of pancreatic cancer by degrading HTR1D.

### LncRNA HOXA10-AS, which was negatively correlated with miR-340-3p and positively correlated with HTR1D in PCs, could directly interacted with miR-340-3p

**Fig 7:**
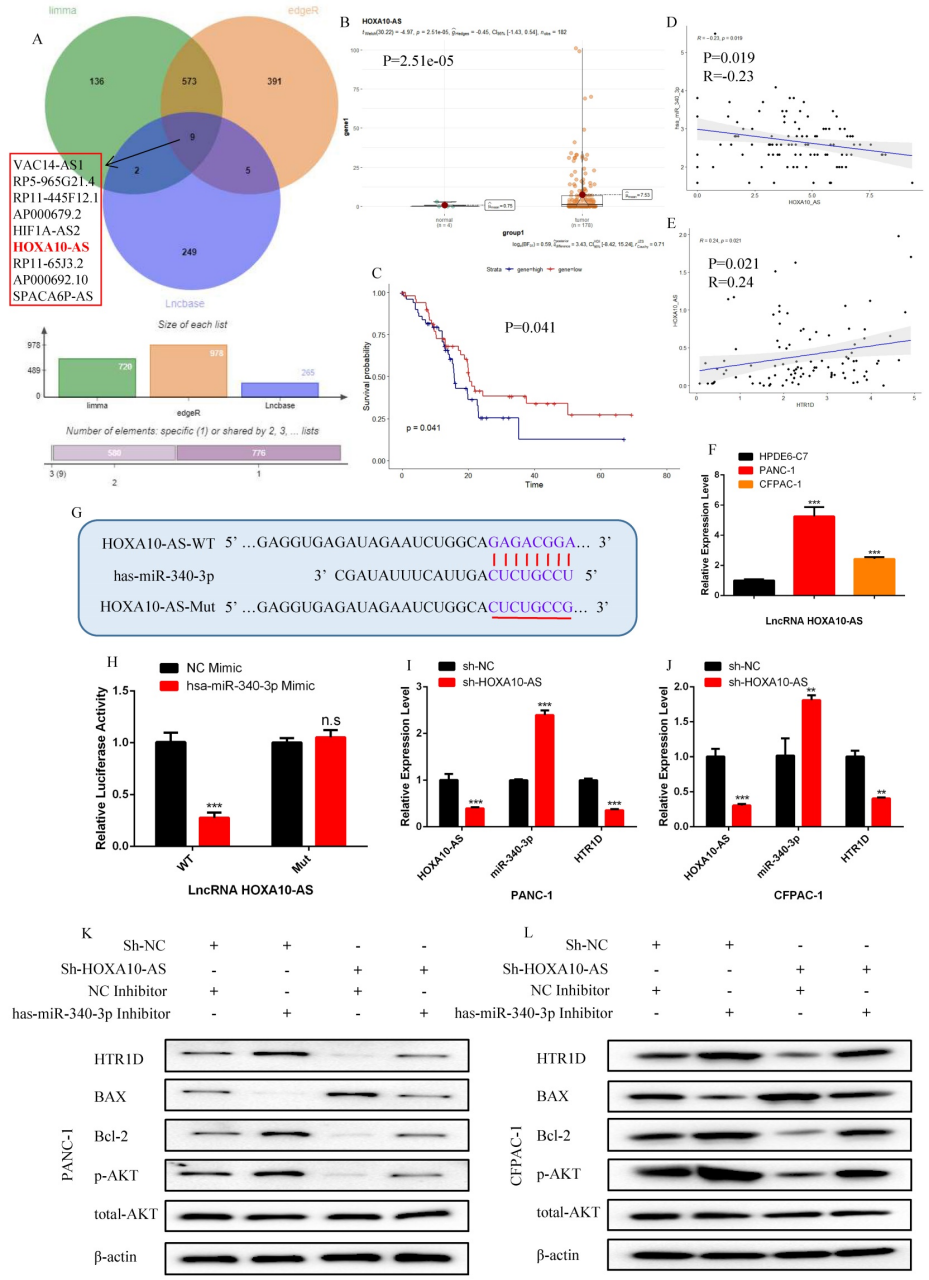
LncRNA HOXA10-AS is negatively correlated with the expression of hsa-miR-340-3p and positively correlated with the expression of HTR1D in pancreatic cancer. **a** The venn diagram for the intersection of the upstream regulatory LncRNAs of hsa-miR-340-3p predicted by Lncbaset database and the differentially expressed LncRNAs calculated by different R packages in TCGA liver cancer samples. **b-c** The expression difference and survival analysis curve of LncRNA HOXA10-AS using RStudio based on the bioinformatics analysis of TCGA data. **d-e** Correlation analysis of the expression of HTR1D/hsa-miR-340-3p/HOXA10-AS in TCGA pancreatic cancer samples. **f** The mRNA expression level of LncRNA HOXA10-AS was detected by qPCR in PANC-1 and CFPAC-1. **g-h** The dual luciferase reporter gene assay was performed to verify the interaction between LncRNA HOXA10-AS and hsa-miR-340-3p. **i-j** The effect of decreasing LncRNA HOXA10-AS on the expression level of HTR1D and hsa-miR-340-3p in each cell line by qPCR. **k-l** Western blot was performed to detect designated proteins in each cell line transfected with specific plasmids. Each experiment was performed three times independently and results are presented as mean ± s.d. Student's t-test was used to analyze the data. (^*^p < 0.05; ^**^p < 0.01; ^***^p < 0.001)

To continue the identification of the upstream regulatory molecules of the miR-340-3p/HTR1D axis, we took the intersection of the predicted lncRNAs from LncBase database and the differentially expressed lncRNAs from TCGA, and obtained 9 candidate lncRNAs (Fig. 7a).

Among all the candidate lncRNAs, only lncRNA HOXA10-AS and lncRNA AP000679.2 had statistically significant effects on the survival of pancreatic cancer patients (Fig. S1i-p). Unfortunately, lncRNA AP000679.2 had no significant correlation with the miR-340-3p/HTR1D axis (data not shown), so we finally choose lncRNA HOXA10-AS as the research candidate. As shown in Fig. 7b-f, we found that HOXA10-AS was upregulated and correlated with the miR-340-3p/HTR1D axis in pancreatic cancer, as well as had a significant impact on patient survival. Then, we constructed related reporter gene plasmids and performed a dual luciferase reporter gene experiment. The results showed that the fluorescence activity of the group cotransfected HOXA10-AS-WT and miR-340-3p mimic was significantly reduced compared with that of the other groups (Fig. 7g-h). In addition, qPCR results revealed that the expression level of miR-340-3p was upregulated after eliminating HOXA10-AS, and that HTR1D was downregulated accordingly (Fig. 7i-j).

### LncRNA HOXA10-AS, as a ceRNA, competed with HTR1D and miR-340-3p to promote the malignant progression of pancreatic cancer in vitro

**Fig 8:**
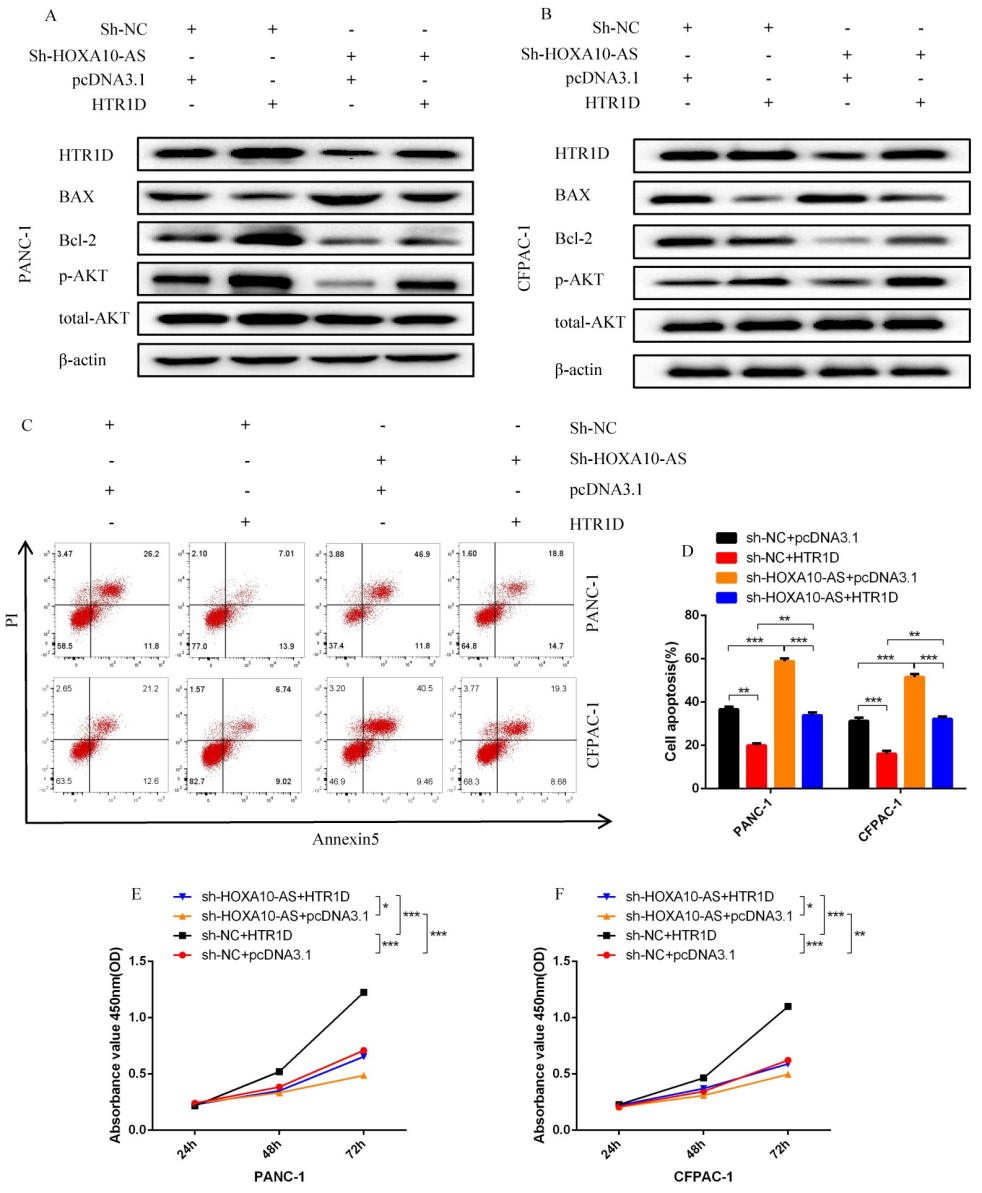
LncRNA HOXA10-AS inhibits the apoptosis of PCs in vitro. **a-b** Western blot was performed to detect designated proteins in each cell line transfected with specific plasmids. **c-d** Flow cytometry analysis was applied to detect cell apoptosis for two cell lines transfected with corelated plasmids. d revealed quantification of the data shown in c. **e-f** CCK8 was performed to evaluate the cell viability of PANC-1 (e) and CFPAC-1 (f). Each experiment was performed three times independently and results are presented as mean ± s.d. Student's t-test was used to analyze the data. (^*^p < 0.05; ^**^p < 0.01; ^***^p < 0.001)

**Fig 9:**
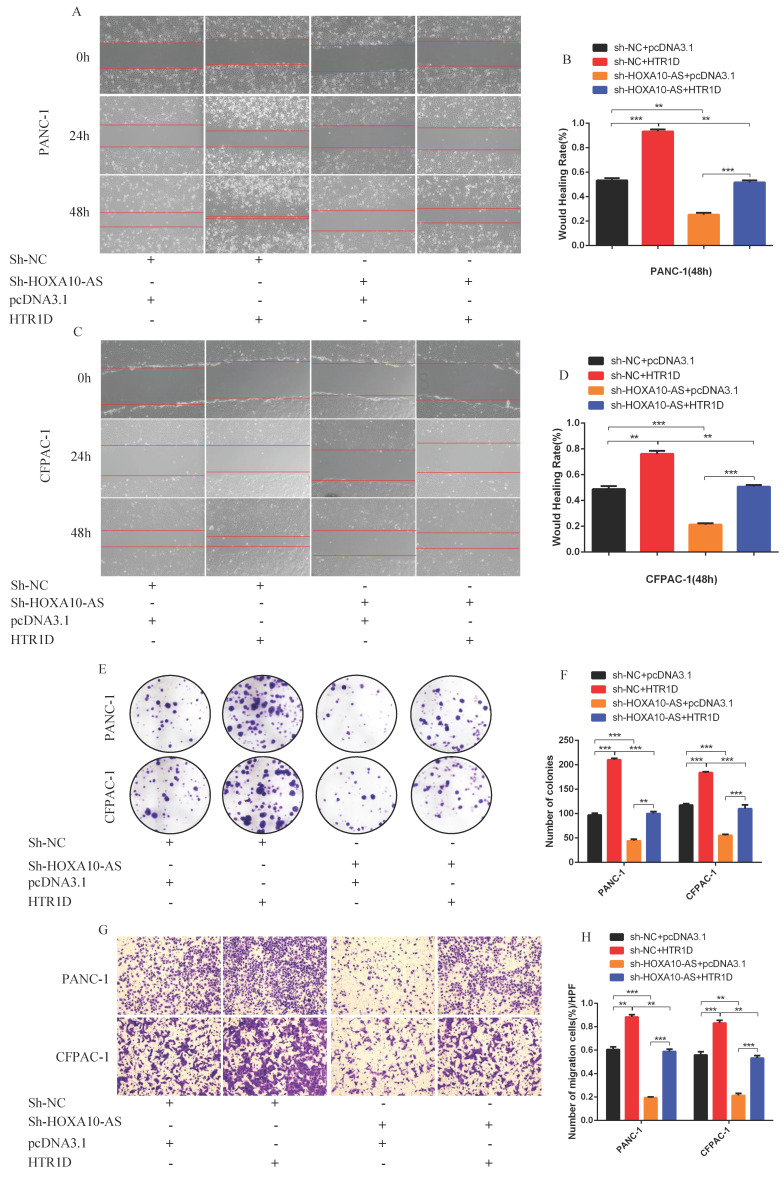
LncRNA HOXA10-AS promotes the migration and proliferation of pancreatic cancer cells in vitro. **a-d** Representative images of wound healing assay for two cell lines transfected with corelated plasmids. b and d revealed quantification of the data shown in a and c. **e-f** Representative images of colony formation assay for PANC-1 and CFPAC-1. **g-h** Representative images of the migration ability of PANC-1 and CFPAC-1 by Transwell assay. Each experiment was performed three times independently and results are presented as mean ± s.d. Student's t-test was used to analyze the data. (^*^p < 0.05; ^**^p < 0.01; ^***^p < 0.001)

**Fig 10:**
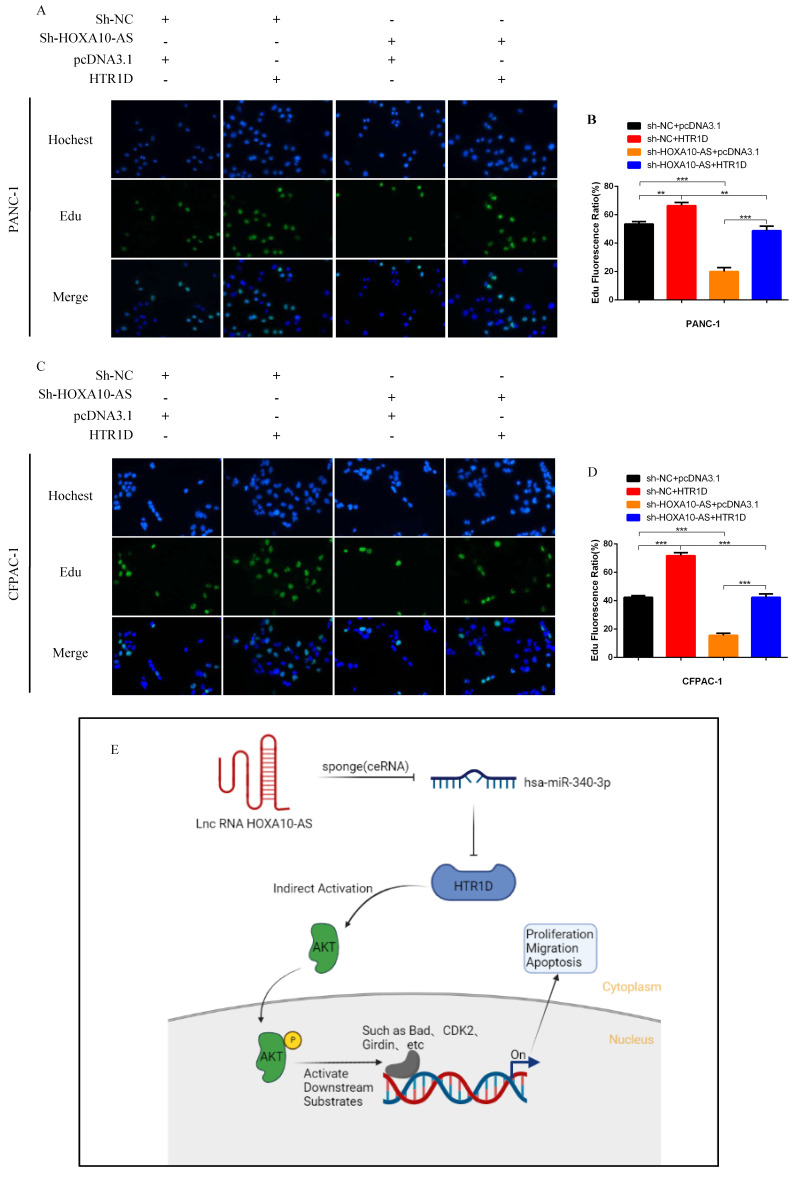
Schematic diagram. **a-d** EdU incorporation assay was performed to detect the proliferation of PANC-1 (a) and CFPAC-1 (c). b and d revealed quantification of the data shown in a and c. **e** The schematic diagram for the overall idea of this research. Each experiment was performed three times independently and results are presented as mean ± s.d. Student's t-test was used to analyze the data. (^*^p < 0.05; ^**^p < 0.01; ^***^p < 0.001)

To test whether lncRNA HOXA10-AS promotes the progression of pancreatic cancer through the miR-340-3p/HTR1D axis, a series of gain- or loss-of-function assays were performed. According to the WB results, we found that lessened lncRNA HOXA10-AS cut down the expression level of HTR1D, Bcl-2, p-AKT, and conversely increased Bax. However, either decreased miR-340-3p or elevated HTR1D attenuated the above effects of reduced HOXA10-AS (Fig. 7k-l, Fig. 8a-b). Then, we performed flow cytometry and found that the knockdown of lncRNA HOXA10-AS obviously increased the apoptosis ratio of PCs, which was neutralized by upregulated HTR1D (Fig. 8c-d). The CCK-8 results showed that the downregulation of lncRNA HOXA10-AS reduced the absorbance of tumor cells at 450nm, whereas increased HTR1D reversed this phenomenon (Fig. 8e-f). Knocking down HOXA10-AS significantly reduced the wound healing rate of PCs, while overexpressing HTR1D eliminated the above effect (Fig. 9a-d). Similarly, the results of colony formation assays and Transwell assays showed that downregulated HOXA10-AS had a significant inhibitory effect on the colony number and migration rate of PCs. Nevertheless, we overexpressed HTR1D after knocking down HOXA10-AS, and we finally found that the number of colonies and the migration rate rebounded (Fig. 9e-h). In addition, we performed EdU assays and finally found that, consistent with the previous trend, knocking down HOXA10-AS led to a remarkable decrease in the fluorescence ratio, while overexpressing HTR1D would increase the fluorescence ratio (Fig. 10a-d). Finally, we generated a brief mechanism diagram through the Biorender website (https://biorender.com/) (Fig. 10e). All in all, we found that lncRNA HOXA10-AS could function as a ceRNA to sponge miR-340-3p to regulate HTR1D, which promoted the progression of pancreatic cancer.

## Discussion

Pancreatic cancer is one of the most malignant tumors at present, and its pathogenesis is poorly understood, which leads to the limitations in its treatment 30. Therefore, exploring novel biomarkers for early detection and treatment is of great significance and will provide us with powerful ideas and strategies to overcome pancreatic cancer. With the improvement of scientific research technology and the continuous research, increasing evidences have shown that the ceRNA regulatory network plays a crucial role in the occurrence and development of diseases, especially malignant tumors such as pancreatic cancer 31. For example, Lei S et al. found that lncRNA 00976 could promote pancreatic cancer progression by competitively sponging miR-137 to upregulate OTUD7B through the EGFR/MAPK pathway 32. Zhang H et al. found that lncRNA PSMB8-AS1 contributed to the progression of pancreatic cancer by modulating the miR-382-3p/STAT1/PD-L1 axis 33. At present, there are many similar studies. Although many molecular targets have been found to be involved, there are still very few that can truly be applied for clinical diagnosis and treatment. Therefore, there is an urgent need to discover increasingly useful molecular biomarkers to improve the efficiency of the diagnosis and treatment of pancreatic cancer.

In this work, the three components of HOXA10-AS/miR-340-3p/HTR1D axis were all reported for the first time to be significantly related to the occurrence and development of pancreatic cancer. Although it is not clear what the application prospects of these three molecules are, this study provides a theoretical basis for their application in the diagnosis and treatment of pancreatic cancer. We have proven that HOXA10-AS/miR-340-3p/HTR1D axis can regulate the progression of pancreatic cancer, but it is still unclear whether the key molecule HTR1D affects the PI3K-AKT signaling pathway directly or indirectly, so more mechanistic studies are needed. In addition, we have not yet further explored what other transcription factors are involved in the upstream regulation of this axis. We will address these gaps in future works. In the present research, we performed a series of in vitro and in vivo assays to clarify the importance of HTR1D in pancreatic cancer, but we did not construct knockout mice or tumor orthotopic transplantation models to provide stronger evidence due to funding and other reasons, but these approaches are worth further exploration.

Even though we reported the involvement of HOXA10-AS/miR-340-3p/HTR1D axis in the ceRNA network of this study, this phenomenon didn't mean that there were no other ncRNAs participating in the regulation of HTR1D and thus affected the progression of pancreatic cancer. Other ncRNAs involved in the regulation of HTR1D in the development of pancreatic cancer still need to be further explored. Although this was the first time that lncRNA HOXA10-AS and miR-340-3p were reported to be relevant to the progression of pancreatic cancer, there exsited other studies reporting that lncRNA HOXA10-AS promoted the proliferation of oral cancer 34 and was also a new oncogene in gliomas 35. LncRNA HOXA10-AS could also promote the progression of lung adenocarcinoma through the Wnt/β-catenin signaling pathway 36. In addition, some studies demonstrated that miR-340-3p exerted a major effect on other tumors. For instance, Yan L et al. found that lncRNA H19, as a ceRNA, could sponge miR-340-3p to promote epithelial-mesenchymal transition by regulating YWHAZ expression in paclitaxel-resistant breast cancer cells 37. The research team from China Medical University declared that the miR-340-3p-HUS1 axis inhibited the proliferation and migration of lung adenocarcinoma cells 38. Another study pointed out that the circular RNA circPPFIA1 promoted disease progression by sponging miR-340-3p and regulating ELK1 expression in laryngeal squamous carcinoma 39. Whether HTR1D also plays pivotal roles in other kinds of tumors is still unknown and remains to be discovered by interested research groups.

In summary, although this work initially substantiated that the lncRNA HOXA10-AS/miR-340-3p/HTR1D axis was involved in the occurrence of pancreatic cancer, more direct evidence was still needed to further confirm our conjecture. Meanwhile, we need to perform more experiments to clarify the specific underlying molecular mechanism regarding this ceRNA network. Cancer is the most well-studied disease worldwide, the diagnosis and treatment of tumors is still a major obstacle. Therefore, the discovery of increasingly practical and useful biomarkers and clarification of the molecular mechanisms are very important for developing effective tumor treatments. We believe that people will find the key to tumor treatment in the near future.

## Supplementary Material

Supplementary figure and table.

## Abbreviations

PCs: Pancreatic cancer cell lines
HTR1D: Hydroxytryptamine receptor 1D
ceRNA: Competing endogenous RNA
WB: Western blotting
IHC: Immunohistochemistry
CRC: Colorectal cancer
ncRNA: Noncoding RNA
PI: Propidium iodide
PBS: Phosphate-buffered saline
UTR: Untranslated region
GEPIA: Gene Expression Profiling Interactive Analysis
GO: Gene Ontology
KEGG: Kyoto Encyclopedia of Genes and Genomes
TBST: Tris-buffered saline with 0.1% Tween20
cDNA: Complementary DNA
CCK-8: Cell Counting Kit-8
MRE: MicroRNA response element
