# Supplementary material for: HTR1D functions as a key target of HOXA10-AS/miR-340-3p axis to promote the malignant outcome of pancreatic cancer via PI3K-AKT signaling pathway
Source: Int J Biol Sci. 2022 May 27;18(9):3777–94. doi: 10.7150/ijbs.70546 (PMC9254475; doi:10.7150/ijbs.70546)
Supplement: Supplementary file 1 — Supplementary figure and table. [file ijbsv18p3777s1.pdf]

Fig.S1 The analysis of TCGA data and Screening for target genes. **a-d** Survival analysis of the remaining 4 hub genes except HTR1D. **e-f** The heatmap for differentially expressed miRNAs and LncRNAs of paired pancreatic cancer in TCGA database. **g-h** The volcano map for differentially expressed miRNAs and LncRNAs of paired pancreatic cancer in TCGA database. i-p Survival analysis of the remaining 8 selected LncRNAs except LncRNA HOXA10-AS.

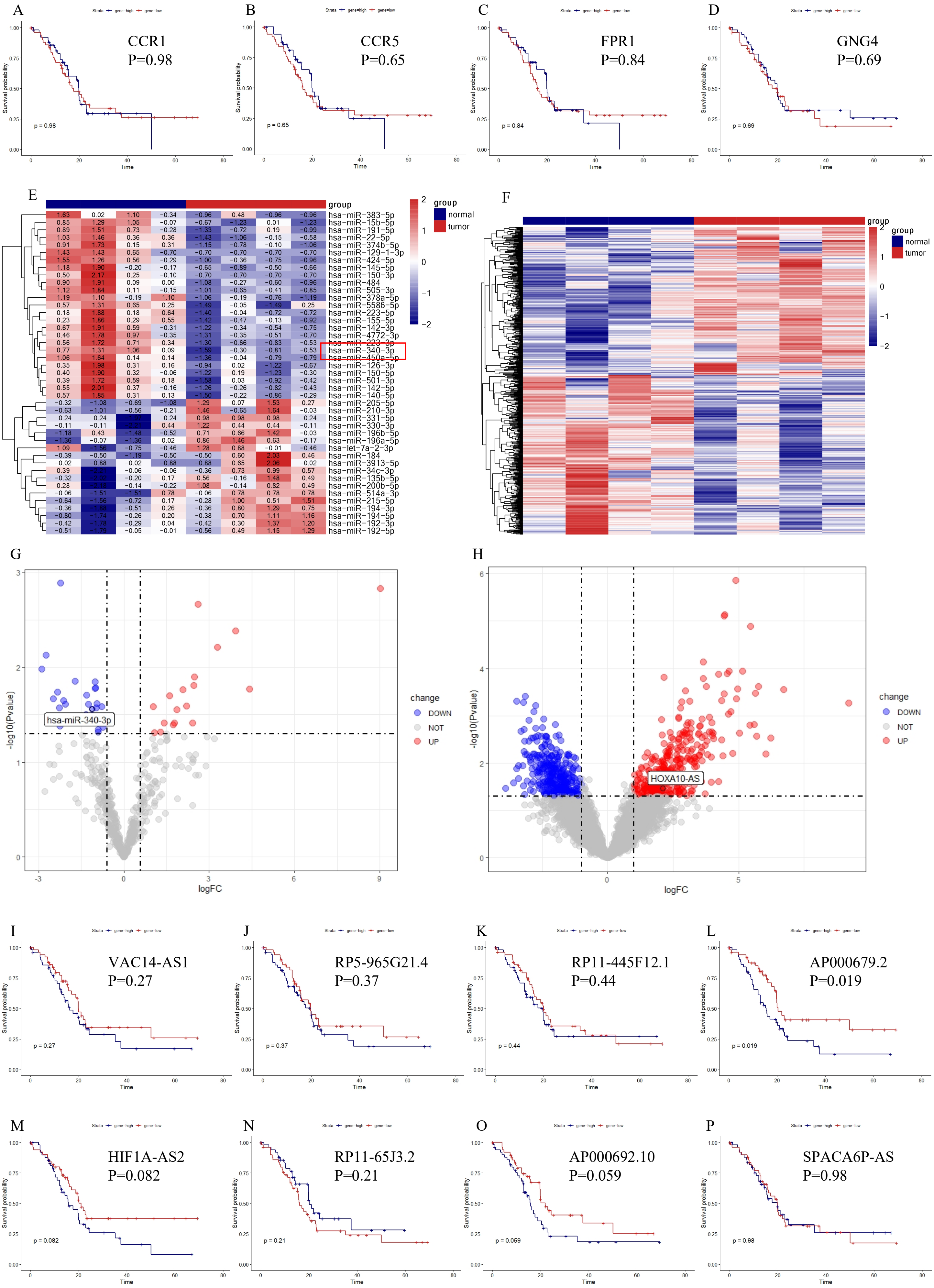
A
CCR1
P=0.98
p = 0.98
B
CCR5
P=0.65
p = 0.65
C
FPR1
P=0.84
p = 0.84
D
GNG4
P=0.69
p = 0.69
Strata gene=high gene=low
Survival probability
Time
E
group
normal
tumor
hsa-miR-383-5p
hsa-miR-15b-5p
hsa-miR-191-5p
hsa-miR-22-5p
hsa-miR-374b-5p
hsa-miR-129-1-3p
hsa-miR-424-5p
hsa-miR-145-5p
hsa-miR-150-3p
hsa-miR-484
hsa-miR-505-3p
hsa-miR-378a-5p
hsa-miR-5586-5p
hsa-miR-223-5p
hsa-miR-155-5p
hsa-miR-142-3p
hsa-miR-4772-3p
hsa-miR-223-3p
hsa-miR-340-3p
hsa-miR-450a-5p
hsa-miR-126-3p
hsa-miR-150-5p
hsa-miR-501-3p
hsa-miR-142-5p
hsa-miR-140-5p
hsa-miR-205-5p
hsa-miR-210-3p
hsa-miR-331-5p
hsa-miR-330-3p
hsa-miR-196b-5p
hsa-miR-196a-5p
hsa-let-7a-2-3p
hsa-miR-184
hsa-miR-3913-5p
hsa-miR-34c-3p
hsa-miR-135b-5p
hsa-miR-200b-5p
hsa-miR-514a-3p
hsa-miR-215-5p
hsa-miR-194-3p
hsa-miR-194-5p
hsa-miR-192-3p
hsa-miR-192-5p
F
group
normal
tumor
G
change
DOWN
NOT
UP
hsa-miR-340-3p
-log10(Pvalue)
logFC
H
change
DOWN
NOT
UP
HOXA10-AS
-log10(Pvalue)
logFC
I
VAC14-AS1
P=0.27
p = 0.27
J
RP5-965G21.4
P=0.37
p = 0.37
K
RP11-445F12.1
P=0.44
p = 0.44
L
AP000679.2
P=0.019
p = 0.019
M
HIF1A-AS2
P=0.082
p = 0.082
N
RP11-65J3.2
P=0.21
p = 0.21
O
AP000692.10
P=0.059
p = 0.059
P
SPACA6P-AS
P=0.98
p = 0.98

Supplementary Table 1. Primers for qPCR.

Primer name	Primer sequence(5'-3')
GAPDH	AGCCACATCGCTCAGACAC (F)
	GCCCAATACGACCAAATCC (R)
U6	AACGCTTCACGAATTTGCGT (F)
	CTCGCTTCGGCAGCACA (R)
HTR1D	GCCTATACCATCACCCACA (F)
	TCCAGAGCAATGACACAGA (R)
1#Sh-HTR1D	GCATCTCTGTGTCATTGCTCT
2#Sh-HTR1D	GCCAAATCTTGTGTGACATCT
miR-340-3p	ACACTCCAGCTGGGTCCGTCTCAGTTACTT (F)
	TGGTGTCGTGGAGTCG (R)
340-3p mimic	UCCGUCUCAGUUACUUUAUAGC
	UAUAAAGUAACUGAGACGGAUU
340-3p inhibitor	GCUAUAAAGUAACUGAGACGGA
LncRNA HOXA10-AS	CCCAGTAAGCCAAAGTCAAGCC(F)
	CTGAGGTCAATGGTGCAAAGG(R)
Sh-LncRNA HOXA10-AS	GTTCTGGTGCTGCCCGCGAAGGGCTGCCT
